# Minding the Gap: Leveraging Mindfulness to Inform Cue Exposure Treatment for Substance Use Disorders

**DOI:** 10.3389/fpsyg.2021.649409

**Published:** 2021-03-22

**Authors:** Christine Vinci, Leslie Sawyer, Min-Jeong Yang

**Affiliations:** ^1^Department of Health Outcomes and Behavior, Moffitt Cancer Center, Tampa, FL, United States; ^2^Department of Psychology, University of South Florida, Tampa, FL, United States

**Keywords:** cue exposure, mindfulness, extinction, substance use disorder, treatment

## Abstract

Despite extinction-based processes demonstrating efficacy in the animal extinction and human anxiety literatures, extinction for substance use disorders (SUD) has shown poor efficacy (i. e., cue exposure treatment [CET]). Reasons for this lack of success include common threats to extinction, such as renewal and reinstatement. In recent decades, research on mindfulness for SUD has flourished, and a key aspect of these mindfulness-based interventions includes teaching individuals to stay present with whatever experience they have, even if unpleasant, without trying to change/escape/avoid it. Similarly, CET teaches individuals to not escape/avoid conditioned responses (e.g., craving) by engaging in drug use behavior. This paper discusses how mindfulness-based research and practices could positively influence CET through future research (e.g., Could mindfulness practice attenuate renewal? Might mindfulness training + CET enhance the ability to extinguish the most salient or motivational cues?), with the long-term goal of improving SUD treatment.

## Introduction

The National Survey on Drug Use and Health reported that 21.5 million American adults met diagnostic criteria for a substance use disorder (SUD) in 2018 (USDHHS, 2019). Despite the deleterious effects caused by SUDs, <15% of diagnosed individuals receive treatment; ongoing substance use confers substantial individual, societal, and economic burden (Grant et al., 2015). The National Institute of Drug Abuse (NIDA) reported that abuse of tobacco, alcohol, and illicit drugs costs in excess of $740 billion annually due to crime, lost work productivity, and healthcare (National Institute of Drug Abuse, 2017).

Extinction-based approaches for SUDs have a strong theoretical background within the animal literature, and much research in humans has explored extinction as a treatment modality, often called cue exposure treatments (CETs). Despite decades of research, little progress has been made in addressing the inherent limitations of CET that seem to hinder its long-term efficacy. In this paper, we consider how mindfulness might inform cue exposure, given the natural overlap in both approaches regarding therapeutic implications of non-escape/avoidance processes (often conceptualized as acceptance of the present moment in the mindfulness literature). Findings from existing studies on mindfulness for SUD suggest numerous ways in which mindfulness-based strategies may positively inform extinction-based processes, although very little empirical research has been conducted in this area [note that Treanor (2011) discussed this topic in reference to anxiety disorders; Carmody et al., 2009; Hölzel et al., 2011 also both discuss extinction-based processes in the larger context of mindfulness]. To date, the potential value and application of mindfulness-based approaches regarding CET for SUD has not been discussed. This paper aims to bridge the broader literatures of mindfulness and cue exposure for SUD, with an emphasis on recommendations for future research that might advance SUD treatment.

## Extinction

### Classical Extinction and Its Vulnerabilities

Given that conditioning has been posited to play a critical role in addiction (Wise and Koob, 2014), it would follow that extinguishing these classically and operantly conditioned responses would lead to effective treatment for SUD. For example, if an ashtray [conditioned stimulus (CS)] is repeatedly presented, without allowing the individual to smoke a cigarette [thus, the absence of nicotine or unconditioned stimulus (US)], then craving and/or subsequent drug administration would theoretically extinguish. Consequently, the repeated presentation of the CS extinguishes cue reactivity [a measure of craving, the conditioned response (CR)] which, in turn, is theorized to result in decreased motivation for drug use (Carter and Tiffany, 1999).

However, once classical extinction learning occurs, it is vulnerable to at least two well-documented threats, spontaneous recovery and reinstatement (Chance, 2013). Spontaneous recovery involves the return of the CR (e.g., craving) after the passage of time following its extinction. As such, exposure to the CS (e.g., drug-related stimulus) will again elicit the CR. Alternatively, reinstatement may occur following extinction of the CR if the organism encounters the unconditioned stimulus (US), such as a drug—without the CS—and the CR (e.g., craving) returns. Both spontaneous recovery and reinstatement are also examples of an overarching vulnerability of classical extinction, context dependence. Context-dependent responding has been demonstrated in various conditions that comprise the context, such as the passage of time (spontaneous recovery), stimulus encounter (reinstatement), or in other situations in which the current context promotes retrieval of conditioning as opposed to extinction (Bouton and Swartzentruber, 1991).

### Operant Extinction and Its Vulnerabilities

Operant extinction involves withholding reinforcement for behavior (e.g., drug use) that was previously reinforced, such that the behavior eventually will cease (e.g., Chance, 2013). Extinction is often context dependent, in that extinction learning may not generalize to other contexts outside of the environment where the extinction trials occurred (both for classical and operant extinction). Renewal and resurgence are two processes that demonstrate the context-dependency of extinction learning. Renewal involves the recurrence of behavior following extinction when the organism returns to an environment where the behavior was previously reinforced (Bouton and Bolles, 1979). Resurgence can also threaten extinction such that increases in previously reinforced responding can occur during the extinction of another more recently reinforced response (Doughty and Oken, 2008). Critically, conditioned responses readily generalize, whereas extinction learning exhibits context discrimination (Bouton and Bolles, 1979). Furthermore, the majority of preclinical studies used to inform CET have relied upon extinction of operant responding, which could be why animal research has not translated into treatment gains. Thus far, only two studies have investigated cue extinction in the absence of operant extinction—conditions that mimic CET—for the reduction of drug seeking (Kim et al., 2014; Madsen et al., 2017). Further preclinical investigation is warranted for the elucidation of processes that could enhance the efficacy of CET. As such, this could explain why addictive behaviors can be resistant to extinction (Doughty and Oken, 2008).

### Extinction-Based Treatment for SUD

CET is an extinction-based treatment for SUD. Although CETs can vary in terms of location, duration, and frequency, the general procedure for CET involves the repeated presentation of stimuli associated with drug use in the absence of drug administration with the aim of reducing cue reactivity (e.g., Carter and Tiffany, 1999). Reduction of cue reactivity is posited to attenuate subsequent drug use by extinguishing automatized conditioned responses that motivate drug use (Carter and Tiffany, 1999). The cues (e.g., ashtray, lighter, glass of beer) can be presented in numerous ways including photographs, video, audio, *in vivo*, imaginal, virtual, or augmented reality, or as a combination of the preceding exposure methods. Moreover, in addition to external stimuli, exposure to affective cues, such as conditioned emotional states (Stasiewicz and Maisto, 1993; Vinci et al., 2012) or thoughts (Tiffany, 1990), as well as to interoceptive physiological cues (Martin et al., 2010) can be utilized. Measures such as craving, mood, and physiological symptoms are assessed in response to the drug cues, often in comparison to neutral stimuli (Franken et al., 1999).

The cues utilized in CET attain association with drug use (as CSs) through prior classical conditioning. Thus, the rationale for CET is that the presentation of the CS without the US will eventually extinguish the CR (Conklin and Tiffany, 2002). Extinction of the CR is theorized to reduce the motivation for ensuing drug use, given that CRs can include craving, negative affect, and withdrawal symptoms, all of which can motivate drug seeking (Carter and Tiffany, 1999). If the CR that motivates drug seeking is extinguished, then operant behavior (drug use) may cease.

The general mechanism by which the CR is thought to reduce was originally posited to occur via habituation, which is a learning process that results in diminishment of the response after repeated or extended exposure to the CS. Though habituation-based models are still well accepted, more recently, support has been garnered by inhibitory learning approaches as an augmentation to exposure treatment, particularly for the treatment of refractory anxiety and phobia (Craske et al., 2008, 2014; Weisman and Rodebaugh, 2018). Maximization of inhibitory learning during exposure is central to these treatments. Clinical approaches that maximize inhibitory learning during exposure, such as expectancy violation, deepened extinction, occasionally reinforced extinction, removal of safety signals (prevention of their use), stimulus variability, extinction retrieval cues, extinction in multiple contexts, and memory reconsolidation have all been utilized to augment exposure therapy for the treatment of anxiety-related disorders (Craske et al., 2014). These approaches have been shown to increase the effectiveness of anxiety exposure treatment, and those that are applicable to the treatment of SUDs, such as deepened extinction, extinction retrieval cues, and multiple contexts will be addressed later in this paper.

### Limited Efficacy and Effectiveness for the Treatment of SUDs

Extinction-based treatment (i.e., exposure therapy) has been shown to be highly effective in other disorders, particularly phobias and anxiety disorders (e.g., McLean et al., 2015). However, while theoretically promising, CET for the treatment of addiction has produced mixed results. CET has shown some positive effects concerning craving reduction and decreases in subsequent drug use (Childress et al., 1986, 1988; O'Brien et al., 1990; Drummond and Glautier, 1994; Franken et al., 1999; Niaura et al., 1999); however, other studies found no effect (McLellan et al., 1986; Dawe et al., 1993, 2002). Moreover, some studies reported increases in craving and drug use following CET (Lowe et al., 1980; Corty and McFall, 1984; Marissen et al., 2007). A meta-analysis conducted by Conklin and Tiffany (2002) and a systematic review by Martin et al. (2010) both concluded that there is no decisive evidence for the use of CET to treat SUD. Explanations for the limited efficacy of CET have generally been associated with processes that hinder extinction (Conklin and Tiffany, 2002). Some common threats to extinction were briefly mentioned earlier and will be discussed in more detail later (renewal effect; spontaneous recovery; reinstatement; failure to extinguish certain cues; resurgence). Additionally, a very practical hindrance to CET is the experience of heightened levels of emotion that can interfere with exposure (Monti and Rohsenow, 1999; Dharmadhikari and Sinha, 2015), which will also be discussed.

### CET and Mindfulness

CET is strongly rooted in learning that drug-related cues no longer predict the drug experience, such that the CR (e.g., craving) can be inhibited when confronted with drug-related cues in the future. This is accomplished by presenting the CS (e.g., drug paraphernalia) over multiple trials/sessions to reduce the CR. Similarly, and as discussed in more detail in the next section, mindfulness-based interventions (MBIs) foster an individual's ability to “sit with discomfort (i.e., craving for the drug),” rather than reacting to it by trying to change, escape, or avoid it. From a neurobiological perspective, there also appears to be overlap in those brain regions beneficially affected by both extinction and mindfulness interventions (e.g., prefrontal cortex; Myers and Davis, 2006; Hölzel et al., 2011; Allen et al., 2012; Bossert et al., 2013; Creswell and Lindsay, 2014; Schuman-Olivier et al., 2020). As such, MBIs may set a stage to facilitate CET by addressing some of the longstanding threats to extinction, which could lead to the development and implementation of stronger SUD treatments.

## Mindfulness

Mindfulness has been defined as the awareness that comes from directing attention to the current moment, without judging that moment as good or bad (Kabat-Zinn, 1994; Shapiro et al., 2006). Mindfulness practices aim to cultivate a sense of nonjudgment and acceptance toward experiences (e.g., emotions, physical sensations), all while maintaining focus on the present moment by recognizing whatever is occurring. Through this process, individuals learn to observe thoughts and emotions, without automatically reacting to them or trying to change them in some way. As such, the person learns to become an observer of experiences, and instead of shifting into autopilot, the individual has gained a broader perspective on the situation and can thoughtfully choose the next course of action. In other words, the salience of the autopilot process is increased, such that this awareness can facilitate purposeful choice.

### Mindfulness and Substance Use

Several MBIs exist for SUD, including Mindfulness-Based Relapse Prevention (MBRP; Bowen et al., 2011), Mindfulness-Based Addiction Treatment (MBAT; Vidrine et al., 2016), Mindfulness Treatment for Smokers (MTS; Davis et al., 2014), Mindfulness Treatment (MT; Brewer et al., 2011), and Mindfulness-Oriented Recovery Enhancement (MORE; Garland, 2013). Systematic reviews and meta-analyses have found MBIs to be effective for reducing craving, negative consequences related to use, and stress; there have been mixed findings on changes in frequency and severity of use (Grant et al., 2017; Li et al., 2017; Sancho et al., 2018). Among smokers attempting to quit, participants who completed an 8-week mindfulness treatment (vs. usual care) demonstrated higher likelihood of abstinence via certain mechanisms, including reduced cue reactivity (Spears et al., 2017). Such results suggest that changes in cue reactivity may underlie MBIs, although replication of these findings is needed.

MBIs for SUD include mindfulness meditations and subsequent discussions with participants about how to utilize mindfulness when experiencing cravings or other unpleasant emotional or physical sensations. Here we describe two examples. For instance, participants may be led through a body scan meditation for 25 min. A discussion of what was noticed during the practice is then conducted (e.g., physical tightness/tension in a particular area, wandering thoughts, tingling) as a way to increase awareness of the physical body and reactions to these experiences (e.g., wanting it to go away). This type of discussion often parallels how an individual experiences a craving to use, and how the body and mind react in those moments. For example, understanding how a craving feels physically in the body early on, before it increases so much that it becomes difficult to manage, is important. This ability to notice, without automatically reacting to, is a core feature of mindfulness programs for SUD. Through the body scan and other mindfulness meditations (e.g., mindfulness of breath, mindfulness of sound), individuals are taught how to heighten their awareness to the present moment, without trying to make it different or change it in some way. Such practices are also relevant to understanding triggers to use that are external to the individual (e.g., other people smoking, coffee). By bringing mindfulness to the decision-making process when interacting with the environment, an individual may choose to respond in a manner that supports their substance use goal (e.g., not spend time with other people who are dinking; drinking tea instead of coffee).

Urge surfing is another mindfulness practice that is meant to be a more concrete experiential exercise of what one experiences during a craving (Bowen et al., 2011). Participants are asked to imagine a situation where they experience a craving, and to allow themselves to notice all sensations (e.g., physical sensations, thoughts related to using) that occur right up until they would typically use the substance. In other words, the individual imagines what happens leading up to using, but then pauses immediately prior to imaginal drug administration. They are encouraged stay with these experiences, without trying to get rid of them in any way. The analogy of a wave is then introduced, and individuals are encouraged to imagine themselves surfing the wave (i.e., craving), staying right on top of it, without being submerged or toppled over by it. Over time, the craving to use a substance will decrease naturally, as does a wave. This type of mindfulness practice (along with several other mindfulness exercises) could be considered a type of exposure/extinction process, where participants are encouraged to stay with discomfort for a period of time, in order for it to dissipate on its own.

### Mindfulness and Mechanisms of Relapse

The existing theoretical and empirical literature on mindfulness proposes that MBIs weaken the association between drug cues (i.e., internal and external) and reactivity through increased awareness/attention, the ability to shift out of “autopilot,” and increased tolerance of unpleasant experiences (Garland et al., 2014; Witkiewitz et al., 2014). Regarding attention, mindfulness interventions cultivate the ability to pay attention to both the external (e.g., sounds, smells) and internal environment (e.g., physical sensations, thoughts) through various mindfulness practices. The ability to maintain attention for a period of time, actively shift attention as needed, and the ability to notice when attention has wandered elsewhere are all active components of mindfulness practices (Shapiro et al., 2006; Chiesa and Serritti, 2010; Chiesa et al., 2011). To date, the existing literature across different populations indicates that mindfulness is associated with various attentional outcomes such as increased attention regulation (Lutz et al., 2008; Chiesa et al., 2011) and enhanced cognitive control (Jha et al., 2007, 2010; Tang et al., 2007; Chambers et al., 2008; Moore and Malinowski, 2009; Chiesa et al., 2011). For example, opioid-using patients had significant reductions in attentional bias to pain cues after receiving a mindfulness-based intervention (MORE), which was not the case in the comparison condition (Garland and Howard, 2013). This finding suggests a decreased reactivity to uncomfortable cues via increased awareness of sensations as a result of MORE, leading to the ability to shift attention to other sensations as needed.

Through increased awareness, mindfulness-based practices weaken the link between internal cues and reactivity by cultivating the ability to shift out of “autopilot,” allowing the individual to intentionally decide how to respond to a given situation/emotion/sensation. As a result, the individual often learns to “stay with” discomfort or unpleasant experiences (e.g., craving, negative affect; Witkiewitz et al., 2014) without reacting, which is particularly relevant to relapse prevention, perhaps most specifically for management of craving and negative affect with implications for extinction-based treatments. Several studies have determined that mindfulness attenuates the relationship between negative affect and substance use behavior (Roemer and Orsillo, 2003; Bowen and Marlatt, 2009; Witkiewitz and Bowen, 2010; Witkiewitz et al., 2011; Adams et al., 2012, 2015).

## Leveraging Mindfulness to Address Existing Limitations of Extinction Processes

Though extinction theory was largely developed and tested in animal laboratories, Conklin and Tiffany (2002) found that extinction procedures shown to be effective in these laboratories have not been applied to CET. Thus, the treatment often fails presumably due to lack of theoretical adherence and perhaps because enhancements to these processes may be needed to confer gains from the non-human animal laboratory to naturalistic environments. Here, we will present each threat to extinction, followed by a discussion of how mindfulness, which may bridge the gap from the experimental animal and human literatures to the naturalistic environment, may inform future research to advance SUD treatment (see Table 1 for a summary of this section). Although we present specific examples for each threat to extinction, it is very likely that a mindfulness-based strategy we discuss for one threat could also be beneficial for another.

### Renewal Effect

A well-documented threat to extinction is the renewal effect (Bouton et al., 2012). If drug use occurred in context A, and then CET occurs in context B (a hospital or treatment setting, for example), then the behavior (i.e., drug use) can renew when the organism returns to context A. Renewal demonstrates the context dependency of extinction learning (Bouton and Brooks, 1993). The ideal way to address renewal would be to expose the individual to as many contexts where drug use took place, in order to extinguish reactivity (e.g., Gunther et al., 1998). However, this approach is likely impossible to achieve within the traditional treatment context (e.g., weekly in-person sessions). Nonetheless, attempts to address renewal have included (1) having participants bring photos of their natural environment into the laboratory/clinic (Conklin et al., 2010); (2) having smokers bring video images of smoking cues into their natural environment (Wray et al., 2011); and (3) providing smokers with “extinction cues” in the laboratory for them to bring into their natural environment (Collins and Brandon, 2002; Stasiewicz et al., 2007; Unrod et al., 2014). None of these approaches have been able to completely mitigate renewal.

When considering how mindfulness might address the issue of renewal, we might first define the “context” that mindfulness is often taking place within. Mindfulness practices usually consist of paying attention to sensations of the physical body, with examples of such practices including breath meditation, body scan (i.e., attending to physical sensations in the body), and urge surfing (Kabat-Zinn, 1994; Bowen et al., 2011). If the substance-using individual experiences craving in response to a physical sensation (e.g., withdrawal symptom) or unpleasant emotion (e.g., anger), mindfulness would encourage that person to attend to those sensations and stay with them. In other words, not trying to avoid or escape them. This parallels what is typically done in extinction paradigms, with the difference being that “context A” is that individual's own mind/body. Previous research has highlighted the potential importance of such interoceptive cues within the cue reactivity/extinction context (Schwarz-Stevens and Cunningham, 1993; Conklin and Perkins, 2005; Acheson et al., 2007), yet research within SUD that has attempted to address interoceptive cues via extinction have been unsuccessful (e.g., Vinci et al., 2012). Even with interoceptive cues, it is unclear how strong the external context vs. internal context may be, and what could really be present here is context (internal) within context (external). If mindfulness is only practiced within the treatment context (external), then renewal may still be a problem. Practicing mindfulness outside of treatment as home practice might be important here. On the other hand, mindfulness may serve as an extinction cue, allowing the individual to utilize it whenever needed, regardless of where it is practiced.

By focusing on the potential of mindfulness to draw attention to the internal environment, we do not want to overlook the importance of the external environment, both as related to cues themselves that trigger craving independent of internal response, but also due to the potential interconnection between internal and external environments. Furthermore, cultural differences may play a role in the salience of the internal vs. external context, particularly for those who have collectivist (vs. individualistic) perspectives (Henrich et al., 2010). Moreover, some individuals' substance use motives may be more focused on reward (vs. relief; Witkiewitz et al., 2019), ultimately placing more attention on the external environment (vs. escaping internally distressing cues). Nonetheless, future research may explore utilizing mindfulness practices within the context of extinction to determine whether these practices attenuate the renewal effect, all while considering potential moderators of these effects.

### Spontaneous Recovery

Spontaneous recovery is the sudden recurrence of a previously extinguished CR (or operant behavior) after the passage of time. Although spontaneous recovery is a well-documented vulnerability of extinction, CET studies have done little to investigate how to mitigate its effects (Conklin and Tiffany, 2002; Martin et al., 2010). In their meta-analysis, Conklin and Tiffany (2002) found that CET sessions do not regularly involve what has been learned from the animal literature about spontaneous recovery mitigation. Animal studies demonstrated that extinction learning is maximized when exposure to a single cue is repeated within session (Berman and Katzev, 1972), and that longer inter-trial intervals (Mackintosh, 1974) and between-session spacing (Rescorla, 1997) allow for spontaneous recovery to occur and then to be re-extinguished. However, contrary to these findings, CET studies generally expose multiple cues within sessions and treatment usually occurs for several days sequentially or with minimal time in between occurrences (Conklin and Tiffany, 2002). Thus, spontaneous recovery remains a problem upon CET cessation. Moreover, the optimal sequence and timing of CET remains an empirical question.

Evidence from MBIs may inform how to optimize spacing and timing issues in CET in order to reduce the likelihood of spontaneous recovery. Although the mindfulness literature has not yet determined the ideal timing/spacing of meditations to produce the greatest effect, within MBIs for addiction, more engagement with the mindfulness practices during treatment is associated with better outcomes, and these studies often take place over an 8-week time span, with participants being instructed to practice mindfulness meditations on a daily basis (Bowen et al., 2011; Brewer et al., 2011; Davis et al., 2014; Vidrine et al., 2016). Thus, participants in these treatments are learning to not escape/avoid unpleasant experiences (i.e., “stay with” discomfort) that may be associated with craving for several weeks. Most CET studies have been much more limited in the number of days/weeks participants engage in cue exposure (Conklin and Tiffany, 2002). Additionally, the mindfulness literature has not systematically evaluated short vs. long meditations spaced over various periods of time. Given spontaneous recovery is based on the passage of time, both the mindfulness and extinction literatures can inform one another to develop research questions that could mitigate this threat to extinction. For example, do short, brief mindfulness exercises spaced out over long periods of time attenuate spontaneous recovery?

### Reinstatement

Reinstatement, the recurrence of the CR when an organism encounters the US in the absence of the CS, as noted by Conklin and Tiffany, is less likely to occur with illicit drugs. However, they raise the point that if another substance of addictive liability is introduced, then the individual may relapse. It may also be the case that substances less associated with addiction may serve as USs. For example, abstinent individuals are likely to encounter certain types of USs in daily life, such as secondhand smoke, alcohol-containing mouthwash, or beverages and medications with trace alcohol content (e.g., kombucha tea, non-alcoholic beer, or some cold remedies). Of these examples, it could be that secondhand smoke is the greatest threat to abstinent smokers because its inhalation is unavoidable due to the unpredictability of its occurrence. Furthermore, secondhand smoke contains nicotine (Office on Smoking and Health (US), 2006), the typical US associated with smoking. However, the US also could be any number of other airborne chemicals intermixed within the smoke. As such, secondhand smoke involves an inundation of possible US exposures. If craving and/or subsequent smoking recurs from this exposure, it would be a demonstration of reinstatement. Furthermore, all stimuli associated with a relapse may then become CSs with the potential to elicit craving/subsequent drug use. However, this effect may be attenuated if the lapse is short. Conklin and Tiffany posit that the benefits of CET will remain following a lapse if it is short in duration. Moreover, subsequent CET, or combining CET with other approaches such as mindfulness, may reduce the impact of the relapse.

The CET literature has investigated various strategies to mitigate reinstatement, with few significant results. For example, if clients undergoing CET continue to use drugs in between sessions or relapse, reinstatement is likely. In their systematic review, Martin et al. (2010) suggested that CET studies conducted during inpatient settings may be protective against clients encountering and/or using drugs (US) while undergoing treatment; however, they found that results were mixed in terms of outcomes. Consequently, they did not conclude that inpatient settings were superior to other locations for CET treatment. As another strategy to minimize reinstatement, they referenced a study by Dawe et al. (2002) during which participants were given a priming dose of alcohol prior to CET treatment; additionally, participants were encouraged to drink small amounts of alcohol between sessions with the assumption that exposure to interoceptive cues would promote CET effectiveness and minimize reinstatement effects. However, results from this study did not support that CET was more effective when combining these approaches.

Literature from MBIs may inform CET regarding strategies to address reinstatement. Recent research has shown that mindfulness was able to slow the progression from lapse to relapse during a cigarette smoking quit attempt (Vidrine et al., 2016). Specifically, participants who completed an 8-week mindfulness group were less likely to progress from lapse to relapse than those in either the cognitive behavioral treatment group or usual care (Vidrine et al., 2016). A likely reason for this finding is that mindfulness-based interventions specifically teach individuals how to “stay with” unpleasant experiences and mindfully choose how to respond next (i.e., not just react). Regarding the progression of lapse to relapse, it is possible that those in the mindfulness condition may have responded with less reactivity when a lapse occurred, resulting in a decreased likelihood of relapse (i.e., reinstatement). Replication is needed, and future research may want to examine whether actively applying mindfulness-based strategies to manage lapse is beneficial, perhaps even as an adjunct treatment to CET.

### Failure to Extinguish Certain Cues

The final threat to extinction that Conklin and Tiffany cite as problematic to CET is the failure to extinguish the most salient or motivational cues. This is particularly problematic for cues with dual properties resulting from classical and operant conditioning. For example, a wine glass could be a CS from its prior association with wine (US) that could then elicit craving (CR). However, the glass could dually serve as a discriminative stimulus, which is a signal for operant behavior to produce a reinforcer. In this way, the wine glass is both a CS from classical conditioning and a discriminative stimulus signaling that drinking (operant behavior) will be reinforced by alcohol effects. Hence, the glass elicits craving AND signals the behavior that will provide reinforcement, which makes it a salient cue for drinking. As Conklin and Tiffany warn, if a CR is extinguished, it may still be the case that the stimulus retains properties that could be motivational for substance use. As such, the operant behavior likely must also be extinguished for the cue to lose all of its reactivity. Regarding the types of cues to extinguish, there still remain empirical questions concerning which cues are the most salient and how many cues must be extinguished for CET to be most effective.

In an attempt to expose participants to the most salient and complex cues, some CET studies have utilized virtual reality (Lee et al., 2003, 2004, 2007; Moon and Lee, 2009). Although this technology is theoretically promising to promote extinction, results have been mixed (Martin et al., 2010). Increased craving relative to static pictorial cues has been induced; however, this study only involved one session (Lee et al., 2003). Additionally, while two studies reported no significant reduction in nicotine craving (Lee et al., 2003, 2004) another reported significant reduction in alcohol urges (Lee et al., 2007). Thus, the effectiveness of extinguishing salient and complex cues with the use of virtual reality remains to be known.

Mindfulness has potential to address these limitations in extinguishing the most salient cues. First, an ability that develops via mindfulness practice is the capacity to actively direct and sustain attention as wanted (Shapiro et al., 2006; Chiesa and Serritti, 2010; Chiesa et al., 2011). It is very possible that over time, the most salient cues (or properties of these cues) would be more evident via mindfulness practices through increased attention, and thus, extinction may be more effective. A two-arm experimental study could be implemented, where half of the participants receive mindfulness training + cue exposure and half receive cue exposure only. Along with determining whether group differences emerge via craving over time, outcomes could include measures to assess both salience of and attention to cues. Assessing whether individuals in the mindfulness + cue exposure condition have greater awareness to internal vs. external cues could also inform questions that were discussed above in the renewal section (e.g., whether some individuals place more salience on the internal vs. external environment).

Second, mindful smoking is a specific example of a mindfulness practice that might address this threat to extinction, although research is needed to fully understand whether that is possible. To date, it has been incorporated into existing MBIs for smoking cessation and has not been empirically validated as a stand-alone treatment component (Davis et al., 2014; Hemenway et al., 2021). During mindful smoking, an individual is asked to only focus on smoking the cigarette and the physical sensations they experience while smoking. They are not to engage in other activities that are distracting or pull their attention away from the act of smoking itself (e.g., texting, driving, and talking). Through this exercise, an individual is still receiving the drug itself, but their relationship to the drug use behavior might be different. For instance, they might notice the aversive properties of smoking, and whether smoking is truly providing what they need in that moment (e.g., long-term stress management). Participants might also attend to “new” cues or sensations that they typically do not notice or fully attend to when smoking (e.g., smell, taste, and breathing). In other words, it is possible that an exercise like mindful smoking is actually addressing the most salient and plentiful cues which, if aversive, may indeed lead to extinction of the operant behavior, thus reducing or extinguishing subsequent smoking. Nonetheless, future research is needed to determine whether this is actually the case. This mindfulness practice may also be difficult to implement with some substances (e.g., cocaine, opioids).

### Resurgence

Resurgence, though not previously addressed by the CET literature, is another threat to extinction that warrants attention. Resurgence is both a behavioral process and procedure (Doughty and Oken, 2008) and we will focus on the former here. If behavior A is drug use, then as it undergoes extinction, either via a treatment setting or other means of abstinence, behavior B, which may be a therapeutic strategy, is reinforced. If for some reason the strategy is no longer effective, either because it does not meet the patient's needs at the time or from lapses in fidelity or adherence, the individual may experience resurgence (i.e., relapse) of drug use (behavior A). Resurgence could impact CET in that when the cue undergoes extinction, the individual could theoretically revert to drug use. To demonstrate this possibility, consider that drug use was reinforced and then it undergoes extinction due to abstinence; however, drug cues, for example, remain reinforcing. If the cues are then extinguished through CET, the previously reinforced behavior (drug use) may resurge.

To date, no CET studies have explicitly investigated resurgence. Furthermore, resurgence on its own is seemingly less of a threat than the four issues identified by Conklin and Tiffany. However, if resurgence is combined with one threat they discussed, renewal, then the problem becomes more severe. Animal research has shown that when resurgence and renewal are combined, then relapse is substantially more robust than when either is experienced singularly (Kincaid et al., 2015).

One potential way to address resurgence would be to evaluate the combination of CET and various mindfulness-based strategies. For example, if a recently learned response (behavior B), such as one specific mindfulness strategy, undergoes extinction—either because it is not effective in the given situation or because the patient does not employ the particular skill—then the individual may be less likely to experience resurgence of drug use behavior (behavior A) because the combined use of alternative mindfulness practices and CET would be more resistant to resurgence than either one on its own. Ideally, this would provide the individual with multiple options when experiencing a craving, and therefore not only rely on the extinction process to be successful. In other words, the individual would have a range of alternative responses to a craving (e.g., bringing awareness to the sensations of a craving, pausing before making a decision, urge surfing), such that if one option were not reinforcing or effective in the moment, another strategy could be. Thus, having several options likely could be protective against relapse.

One could argue that other treatment approaches could also be combined with CET in this manner. Although this is a possibility, mindfulness-based interventions have demonstrated effects above and beyond the gold standard (i.e., cognitive behavioral therapy) regarding lapse and relapse (e.g., Bowen et al., 2014; Vidrine et al., 2016). For instance, when compared to cognitive-behavioral relapse prevention, MBRP maintained initial treatment gains through the 12-month follow-up on outcomes such as days of substance use and heavy alcohol use (Bowen et al., 2014). One potential reason for this finding is that with continued practice of mindfulness-based strategies over time, individuals in the MBRP group were better able to notice and tolerate discomfort and unpleasant emotions associated with craving. A second reason may be that mindfulness skills are generalizable to real-world situations, including novel situations, that are directly and indirectly related to substance use. Traditional cognitive behavioral treatment, on the other hand, is primarily focused on addressing specific skills related to the substance itself (e.g., removing smoking paraphernalia, drink refusal skills), which could limit potential treatment gains. Thus, future research may want to evaluate whether CET + mindfulness may better prepare an individual to manage craving and possible relapse than CET alone, and therefore address any concerns related to resurgence. To expand on this last point, examination of a combined CET + mindfulness-based intervention might be particularly useful. For instance, it may be valuable to incorporate formal, tailored hierarchies for exposure to substance cues, combined with mindfulness-based exercises to further enhance staying with present moment experiences. These hierarchies would mimic what is traditionally done in exposure-based treatments for anxiety disorders. Very few CET studies have created specific hierarchies (e.g., Dawe et al., 2002), much less include individualized hierarchies. Leveraging recent advances in technology (e.g., augmented reality; Vinci et al., 2020) may allow for the tailoring of such cue hierarchies in the real-world environment and further enhance such an intervention.

### Heightened Emotion

Heightened emotions are both common and expected with CET (Monti and Rohsenow, 1999; Dharmadhikari and Sinha, 2015). Nonetheless, extant research has shown that intense emotion can not only interfere with extinction learning (Maren and Chang, 2006), but can even strengthen the relationship between the CS and CR (Campbell and Jaynes, 1966). Individuals with high baseline levels of distress (pre-treatment) may also struggle to fully benefit from CET due to engagement in avoidance behaviors, for instance (Monti and Rohsenow, 1999). Thus, one could argue that for CET to be effective, a balance is needed between expected, heightened emotion and unmanageable levels of emotion. Practically speaking, the anxiety literature has shown that heightened emotion can also derail treatment, such that a patient may drop out early (Meyer et al., 2014). Clinicians may also struggle, such that they do not offer CET to patients, or, when they do, prematurely end treatment due to high levels of patient distress (Meyer et al., 2014).

Combining CET with other skills-based treatments may aid in the management of heightened emotion that is expected with CET, and indeed, this has been proposed previously (Monti and Rohsenow, 1999; Dharmadhikari and Sinha, 2015). MBIs may hold particular promise, especially if provided before CET. One could hypothesize that engaging in mindfulness exercises that specifically target the management of intense emotions (e.g., urge surfing) prior to beginning CET would be useful. It is also possible that engagement in any mindfulness meditation (e.g., body scan, mindfulness of breath) teaches individuals to stay present with whatever is happening, even if distressing or uncomfortable, and that this may also allow engagement with CET to be more manageable.

**Table 1:** Mindfulness-based recommendations and empirical questions to address threats to extinction.

**Existing threat to extinction**	**Definition**	**Mindfulness-based recommendation**	**Empirical research question**
Renewal effect	If drug use occurs in Context A, but CET occurs in Context B (e.g., treatment facility), drug use will likely re-occur when individual returns to Context A.	During mindfulness practices the “context” is whatever is happening in that moment, which is often focused on the physical body and mind (e.g., noticing and staying with a craving). Exposure to the internal sensations becomes the focus, and therefore the external context is less relevant.	Would utilizing mindfulness practices (by exposing individuals to internal cues) within the context of extinction attenuate the renewal effect?
Spontaneous recovery	The recurrence of a previously extinguished CR (e.g., craving) and/or an operant response, such as substance use, after the passage of time.	Engagement in mindfulness practices are encouraged on a daily basis throughout mindfulness treatment. Learning to not escape/avoid unpleasant experiences that are associated with craving and/or substance use may occur over a period of several weeks.	Do short, brief mindfulness exercises spaced out over long periods of time mitigate spontaneous recovery?
Reinstatement	The recurrence of the CR when the individual encounters the US in the absence of the CS (e.g., secondhand smoke).	Mindfulness has been shown to slow the progression of lapse to relapse among smokers (which was not the case for CET, CBT or usual care). A potential reason for this is that mindfulness treatment cultivates non-reactivity, which might be a key factor in how an individual responds to a lapse.	Replication of the findings that mindfulness promotes quicker recovery from a lapse (i.e., preventing a full relapse) among other drugs of abuse is needed. Does specifically applying mindfulness-based strategies in the post-quit period (perhaps in conjunction with CET) aid in managing lapses and preventing full relapse?
Failure to extinguish certain cues	The failure to extinguish the most salient (classical learning) or motivational cues (operational learning).	Enhancement of attention via mindfulness may increase salience of cues making extinction more effective.	Would mindfulness training + cue exposure enhance salience of and attention to substance use cues?
		Mindful smoking is an exercise that has the potential to change an individual's relationship with smoking a cigarette. Noticing certain experiences (e.g., taste, smell) in this manner could address some of the most salient and/or motivational cues leading to extinction of the operant behavior.	Does mindful smoking result in extinction of craving and/or reductions in smoking behavior?
Resurgence	Relapse to previously extinguished behavior during the extinction of a more recently learned response.	Mindfulness-based interventions would provide individuals with a range of options when experiencing a craving, therefore not relying only on the extinction process to be successful.	Would a combined treatment of CET + mindfulness address concerns about resurgence by providing a multitude of options to address craving when it occurs?
Heightened emotion	High levels of intense, unmanageable emotion may impede CET.	Mindfulness meditations or exercises may directly or indirectly aid in the management of distressing or uncomfortable emotional experiences.	Would providing mindfulness training before CET decrease treatment drop-out and/or increase treatment engagement when compared to CET alone?
			Would clinicians be more likely to provide CET if mindfulness training was offered before CET sessions began?

## Future Directions

Multiple recommendations for future research have been discussed in the previous section and are summarized in Table 1. Primary empirical questions include whether engaging in mindfulness practices may address the renewal effect (e.g., Can the mind/body become the primary environment/context of extinction?). Might short, brief mindfulness exercises spaced out over long periods of time attenuate spontaneous recovery? Does mindfulness training + cue exposure increase the salience of cues when compared to cue exposure only? Could mindful smoking aid in extinguishing some of the most salient and motivational cues through extinction of the operant behavior? Would a combined CET + mindfulness-based intervention address resurgence? Would providing mindfulness training prior to CET impact drop-out, treatment engagement, or clinician's willingness to offer CET? Finally, preclinical models of extinction should distinguish between cue extinction vs. the extinction of operant responding to better inform CET, given that cue extinction, but not operant behavior, has been under-investigated in the animal laboratory (Kim et al., 2014; Madsen et al., 2017).

Another question, perhaps most relevant to a combined CET + mindfulness intervention, is how the rationale for such an intervention would be presented to participants. CET is fairly specific in that the goal is to extinguish craving, whereas mindfulness treatments are not focused on reducing craving *per se*, and more focused on changing one's relationship with distress/craving. Thus, one could argue that the therapeutic effect might be attenuated due to a difference in treatment rationale between CET and mindfulness, creating confusion to participants. Here, study design and target threat to extinction may determine how the study rationale is presented. For example, in a study design where mindfulness precedes CET (as in the case of heightened emotion), mindfulness practice may put an emphasis on learning preparatory skills for managing uncomfortable experiences during CET. It is also possible that a complementary position could be taken, such that the rationale for the two approaches are merged (e.g., presenting cues while encouraging participants to sit with discomfort and to notice what happens). This approach may facilitate sustained attention/exposure which could be beneficial for deeper extinction. Despite the current examples, consideration of how these differences can be reconciled is needed.

Moderating factors should also be considered moving forward, specifically regarding whether some individuals may benefit from these treatment approaches more than others. Such research is consistent with the precision medicine initiative of NIH, where ideally individuals could be provided with interventions that are most likely to help them based on personal characteristics. For instance, one could posit that individuals who endorse avoidance/escapist reasons for use (Grunberg et al., 1999) might particularly benefit, given both CET and mindfulness emphasize non-avoidance. Other factors to consider might be the intensity of a craving in a specific moment, reinforcement history of a substance [e.g., intermittent vs. continuous and/or reinforcement strength (e.g., nicotine vs. heroin)], frequency of use (e.g., cigarette smokers who use throughout the day vs. heroin that is used less frequently), motives for use, and cultural influences. Future research will want to consider how these factors might influence the proposed research questions outlined here.

## Summary

In summary, this paper has addressed many deficits in the effectiveness of CET. The many threats to extinction are posited to be the underlying causes of most of these limitations. And although some of these vulnerabilities have been investigated to improve CET, results are still mixed concerning the lasting impact of CET as a treatment for SUDs (Conklin and Tiffany, 2002; Martin et al., 2010). During the past decade, mindfulness-based strategies for the treatment of SUDs have attained wide empirical support (Bowen et al., 2011; Brewer et al., 2011; Davis et al., 2014; Vidrine et al., 2016). Thus, we have identified mindfulness-based practices that may ameliorate known problems with CET, whether to inform or augment the treatment. Although many of these suggestions are currently theoretically based, we identify future directions in which to study the proposed recommendations. Table 1 summarizes the vulnerabilities to CET and potential mindfulness-based strategies to further evaluate. Given the need to provide effective treatment for SUDs, it is paramount to improve upon our current interventions. We strongly believe that mindfulness can be leveraged through future research to “mind the gap” in the CET literature.

## Author Contributions

CV and LS wrote the first draft of the manuscript. CV, LS, and MJY edited the manuscript. All authors contributed to the article and approved the submitted version.

## Conflict of Interest

The authors declare that the research was conducted in the absence of any commercial or financial relationships that could be construed as a potential conflict of interest.
